# The Microstructural Characterization of Multiferroic LaFeO_3_-YMnO_3_ Multilayers Grown on (001)- and (111)-SrTiO_3_ Substrates by Transmission Electron Microscopy

**DOI:** 10.3390/ma10070839

**Published:** 2017-07-21

**Authors:** Bin Pang, Lei Sun, Xuan Shen, Yang-Yang Lv, Xiao Li, Fei-Xiang Wu, Shu-Hua Yao, Jian Zhou, Shan-Tao Zhang, Y.B. Chen

**Affiliations:** 1 National Laboratory of Solid State Microstructures & Department of Materials Science and Engineering, Nanjing University, Nanjing 210093, China; shasiyico@163.com (B.P.); sunleixwz@126.com (L.S.); lvyangws0801@163.com (Y.-Y.L.); wffx0981@sina.com (F.-X.W.); shyao@nju.edu.cn (S.-H.Y.); zhoujian@nju.edu.cn (J.Z.); stzhang@nju.edu.cn (S.-T.Z.); 2 Shanghai Nanoport, Thermo Fisher Scientific Inc., Shanghai 201210, China; xuan.Shen@fei.com; 3 National Laboratory of Solid State Microstructures & Department of Physics, Nanjing University, Nanjing 210093, China; mg1522050@smail.nju.edu.cn

**Keywords:** multiferroic, multilayers, transmission electron microscopy, domain structure

## Abstract

The microstructure of multiferroic LaFeO_3_-YMnO_3_ (LFO-YMO) multilayers grown on (001)- and (111)-SrTiO_3_ substrates is characterized by the transmission electron microscopy (TEM). Detailed TEM characterization reveals that LFO-YMO multilayers grown on both substrates have clear layer-by-layer morphology and distinct chemical-composition layered structure. The most notable feature is that LFO-YMO multilayers grown on (001)-SrTiO_3_ substrate have three types of domains, while those on (111)-SrTiO_3_ have only one. The multi-/twin- domain structure can be qualitatively explained by the lattice mismatch in this system. The details of the domain structure of LFO-YMO multilayers are crucial to understanding their magnetic properties.

## 1. Introduction

Transition-metal perovskite oxides with the chemical formula as ABO_3_ (A and B are alkaline earth/lanthanum and transition metal elements, respectively) demonstrate the whole spectra of physical properties including metal, dielectric, ferroelectric, ferri-/ferro-/antiferro-magnetism and superconductivity [1]. Recently, much of the scientific interest is focused on multi-functionality in single-phase/composite transition-metal pervoskites [1]. Multi-functional materials provide the playground to explore coupling among different elementary excitations in condensed matter physics [2,3,4]. A typical multifunctionality is multiferroicity [2,3,4]. Though great effort has gone into this field during the past two decades, single-phase multiferroic materials are still quite rare [5]. Stacking the multilayers composed of ferroelectric and ferromagnetic/ferrimagnetic perovskite is a feasible way to achieve the multiferroic property [6,7,8]. Guided by this idea, we designed and synthesized a series of LaFeO_3_-YMnO_3_ (LFO-YMO) multilayers on (001)- and (111)-SrTiO_3_ substrates to obtain multiferroicity [9]. The basic consideration to choose this multilayer is that LFO is an insulating antiferromagnetic, and YMO is a weak-multiferroic, but its magnetism is E-type antiferromagnetism. Combining LFO and YMO, interfacial coupling between Mn and Fe should give rise to the strong ferrimagnetism. In this system, we do observe the co-existence of ferrimagnetism and weak ferroelectricity. The measured ferrimagnetism is attributed to antiferromagnetic coupling between Fe and Mn at the interface [9].

The super-exchange interaction between Mn and Fe in a thin-film configuration has been studied for over two decades. Ueda, et al., firstly synthesized LaFeO_3_-LaMnO_3_ multilayers and studied their magnetic properties. They observed the spin-glass behavior in LaFeO_3_-LaMnO_3_ multilayers grown on (001)-SrTiO_3_ (STO) substrate, but ferromagnetic/ferromagnetic behavior in the same multilayers on (111)-STO [10]. In this work, they have not discussed any microstructure analysis and underlying physical origin. Quite recently, Jia et al. observed a more abstract relationship between magnetism and thicknesses of La_0.7_Sr_0.3_MnO_3_/La_0.7_Sr_0.3_FeO_3_ multilayers grown on (111)-STO [11]. The same group also reported the exchange bias and anisotropic exchange interaction in La_0.7_Sr_0.3_MnO_3_/La_0.7_Sr_0.3_FeO_3_ multilayers grown on (111)-STO [12]. In our previous work, one question is unsolved [9]. The anti-ferromagnetic temperature-dependent magnetization (called spin-glass-like) is observed in LFO-YMO multilayers on (001)-STO substrate. Simultaneously, a clear ferromagnetic-hysteresis loop, with a finite residual magnetization as large as 0.3–0.5 *μ*_B_, is observed in these samples. Two natural questions are: why the ferromagnetic hysteresis loop is measured in the LFO-YMO multilayer grown on (001)-STO substrate? Why does Mn-O-Fe bonding display a “spin-glass-like” behavior when multilayers are grown on (001)-STO substrates? We believe that microstructure characterization on LFO-YMO grown on both (001)- and (111)-STO can provide some hints [13].

To fully understand the measured ferrimagnetism in LFO-YMO multilayers, it is very imperative to characterize the microstructure of LFO-YMO multilayers to answer two important questions. One is whether there is a layer-by-layer morphology in multilayers [13]. In other words, Is the inter-diffusion between LFO and YMO layers negligible or not? The other is whether there is a multi-domain structure in the constituent layers. In the magnetic samples with a multi-domain structure, the measured ferromagnetic signals might come from the non-canceled magnetization in the multi-domain structures [14], rather than from designed interfaces.

Taking these above-mentioned issues into consideration, we systematically characterized the microstructure of LFO-YMO multilayers grown on (001)- and (111)-STO substrates by transmission electron microscopy (TEM). Detailed TEM characterization illustrates that there is a clear layer-by-layer morphology and distinctive chemical-composition layered structure on the LFO-YMO multilayers grown on both substrates. The most noticeable feature of these multilayers is that the multilayers grown on (001)-STO substrates have three-type domain structures, but those on (111)-STO have only one. The multi-/twin- domain structure can be qualitatively explained by the lattice mismatch in this system. Based on microstructural characterizations, we think that the multi-/twin- domain structure of LFO-YMO multilayers leads to the ferromagnetic-like hysteresis loop with finite residual magnetization, rather than the interfacial Mn-Fe super-exchange interaction.

## 2. Results

The details of sample growth and TEM work are summarized in experimental section. In this work, three samples were characterized and they are denoted as sample-I, -II, and -III hereafter. The parameters and synthesized conditions of these samples are summarized in Table 1. 

**Figure 1:**
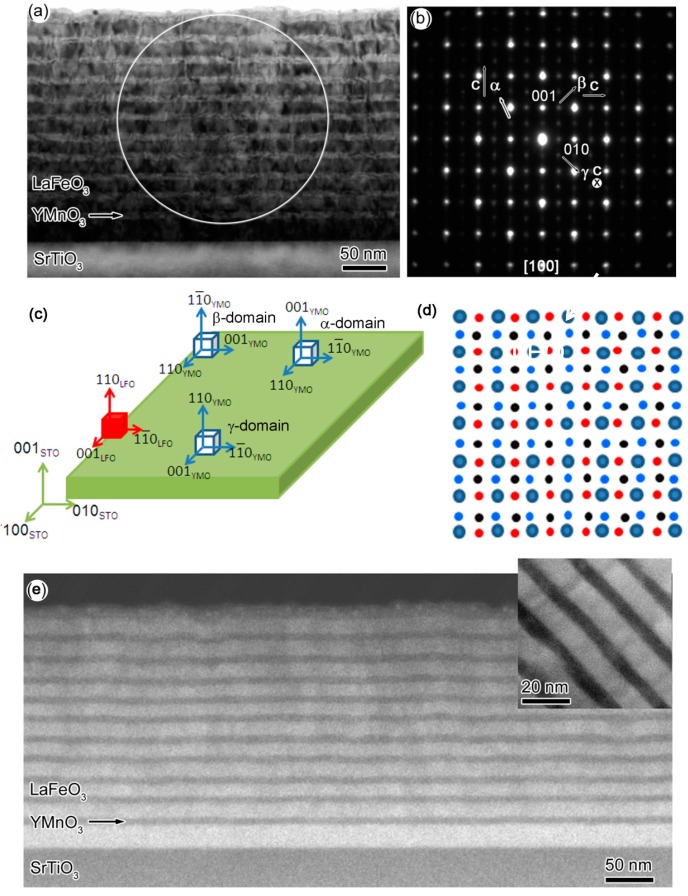
(**a**) The low-magnification transmission electron microscopy (TEM) of LFO-YMO (sample-I) multilayers grown on (001)-SrTiO_3_ (STO) substrate; (**b**) The selected-area-electron-diffraction (SAED) pattern of sample-I. The three weak spots coming from multi-domain structure are highlighted by white arrows. The directions of c-axis in three types of domains of YMO are indicated, as well. The SAED pattern of sample-I are the overlap of four electron diffraction patterns: α-, β-, γ-domain of YMO and [001]-zone axis of LFO. The elongation at the high-order reflections is attributed to splitting of corresponding diffractions; (**c**) is the schematic of three type of crystallographic domain between YMO and LFO in α-, β-, and γ-type of domain configurations; (**d**) is the schematic of SAED pattern of α-, β-, and γ-type of domain configurations. Large violet circles represent the basic reflections of the three types of domains. Small blue, red, and black circles represent the weak reflections coming from α-, β-, and γ-type of domain, respectively; (**e**) The low-magnification high-angle-annular-dark-field (HAADF) image of sample-I. The inset is the high-magnification HAADF image.

Figure 1a shows the low-magnification TEM image of sample-I. The layer-by-layer morphology in this sample is obvious. The white and black contrast layers are YMO and LFO, respectively. Figure 1b is the selected area electron diffraction (SAED) pattern of sample-I that was taken from the area labeled by the white circle in Figure 1a. Apart from the major bright reflections, several weak spots, coming from the multi-domain structure of orthorhombic crystal lattices can also be observed. It should be mentioned that the weak reflection spots do not disappear when we tilted the samples off-zone axis, which rules out the possibility of double diffraction. Specifically, three weak spots, one from the [001]-, and two from the orthogonal [110]-zone axis of orthorhombic perovskite [15], are highlighted by three white arrows. These three domains are called α-, β-, and γ-type domain hereafter. The *c*-axis of α-, β-, and γ-type domains of YMO, as well as (100)-reflection from LFO are also indicated in the Figure 1b. It should be mentioned that LFO layer is mainly composed of γ-type domain analyzed by Fast Fourier Transformation. Multi-domain structure is universally observed in orthorhombic perovskite thin films, such as PrScO_3_ [15], LaFeO_3_ [16], and SrRuO_3_ [17], etc. To elucidate the multi-domain structures in LFO-YMO more clearly, two schematics of orientation-relationships among different domains and simulated electron diffraction patterns are shown in the Figure 1c,d. The orientation relationship between LFO and YMO in α-, β-, and γ-domain can be written as [001] (110) LFO // [110] (001) YMO, [001] (110) LFO // [110] (1$\bar{1}$0) YMO, and [001] (110) LFO // [001] (110) YMO, respectively. The overlapped electron-diffraction pattern composed of [001] and two orthogonal [110] rotated 90° against each other is shown in Figure 1d. Obviously, the simulated electron-diffraction pattern is the same as that in Figure 1b. Figure 1e and its inset are low-magnification and high-magnification high-angle-annular-dark-field (HAADF) images of sample-I, respectively, these images also show clearly layer-by-layer morphology. Apparently, LFO has much brighter contrast than YMO, which is due to the atomic number of La being much larger than that of Y [18].

**Figure 2:**
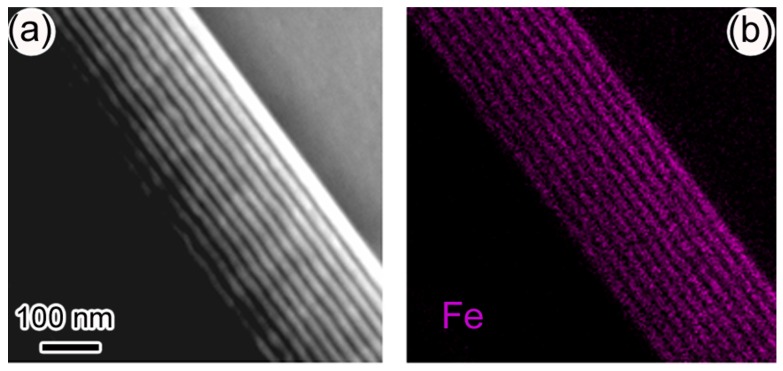

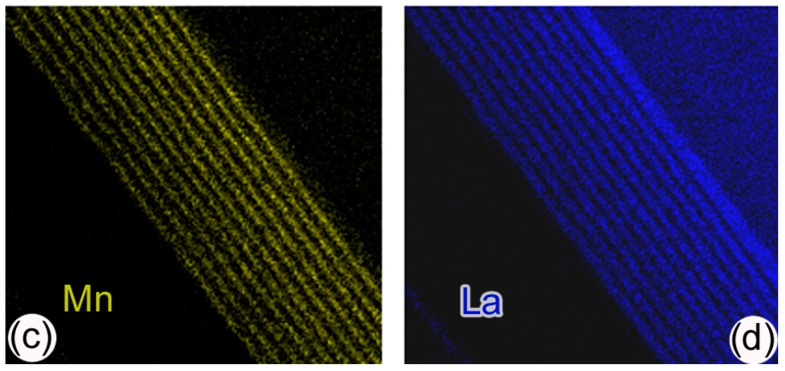
(**a**) HAADF image and (**b**–**d**) are the element mapping images of Fe, Mn, and La of sample-I, respectively.

Figure 2a–d show the HAADF image, as well as energy-filtered TEM images of Fe, Mn, and La of sample-I, respectively. The element mapping images were taken using *L*-peaks of Fe and Mn and *M*-peak of La in electron energy loss spectrum (EELS) [18]. It shows that the element Fe/La and Mn are distributed uniformly across the whole multilayers sample. Using EELS under STEM mode, we did observe the atomically sharp interface between YMO and LFO layers [9].

**Figure 3:**
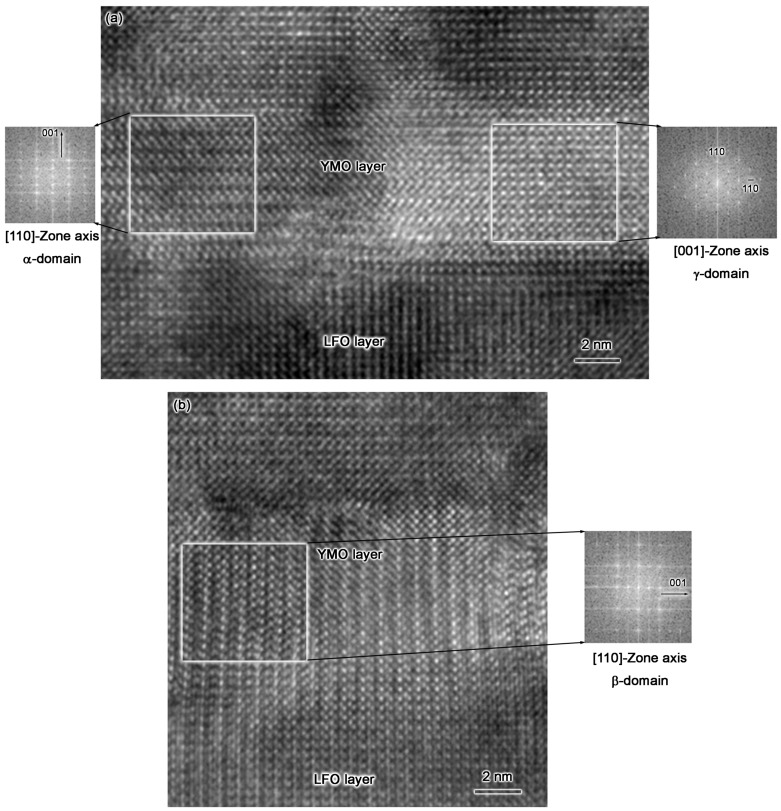
(**a**,**b**) are two typical High-resolution TEM (HRTEM) images of sample-I show that there are multi-domain structures in YMO layers. Three digital Fast Fourier Transformation (FFT) images are depicted in these pictures, as well. The areas used to take FFT images are highlighted by white squares. Obviously, overlapping of three FFT images forms the same pattern as that shown in Figure 1b.

High-resolution TEM (HRTEM) was employed to characterize the multi-domain structures in sample-I. Through our detailed study, the multi-domain structure is mainly formed in the YMO layers. Two typical HRTEM images are depicted in Figure 3a,b. Three different areas with α-, β-, and γ-domains and their corresponding Fast Fourier Transformation (FFT) images are shown in Figure 3, as well. Obviously, there are three types of domain structures in the YMO-layer and a composite pattern of three FFT images is the same as that in Figure 1b. Thus, one can conclude that the multi-domain structure in the YMO layers does exist, as substantiated by both nano-scale FFT and selected area electron diffraction at the micrometer scale. The similar domain structures are also observed in PrScO_3_ films [15]. In addition, we can see from Figure 3 that the interface between YMO and LFO is quite distinguishable.

**Figure 4:**
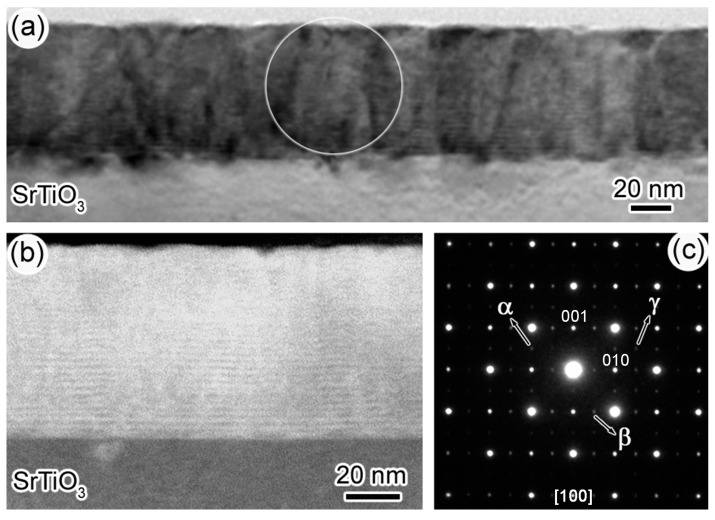
(**a**) The low-magnification TEM image of sample-II; (**b**) The HAADF image of sample-II; (**c**) The SAED pattern of LFO-YMO multilayers. The weak reflection spots coming from three-type domain are highlighted by white arrows.

The low-magnification TEM and HAADF image of sample-II are depicted in Figure 4a,b, respectively. Evidently, this sample also has layered-by-layered morphology. The SAED pattern (Figure 4c) of this sample, taken from area indicated by a white circle, also shows the feature of three-type domain structure.

**Figure 5:**
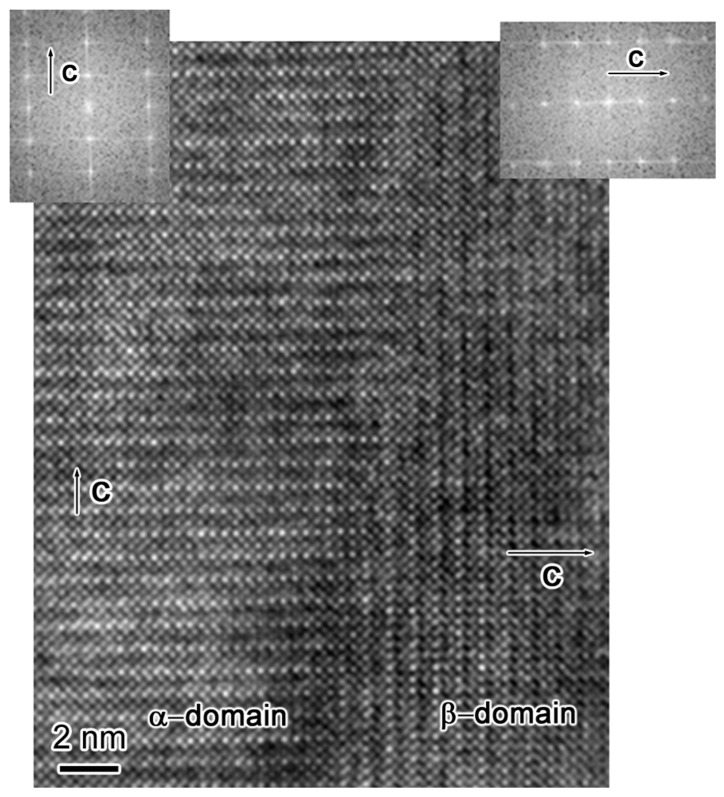
HRTEM image of sample-II shows the two-type domain structure. Left and right insets are the digital Fast Fourier Transformation images of their respective micrograph. The *c*-axis of different domains is indicated by the white arrows.

The multi-domain structure of sample-II is also characterized by HRTEM. Obviously, there are two types of domain structure in Figure 5. The left and right part (of Figure 5) shows the α- and β-domain structure, respectively. The corresponding FFT images are inserted in Figure 5. The c-directions of these two-type domains are labeled in Figure 5.

**Figure 6:**
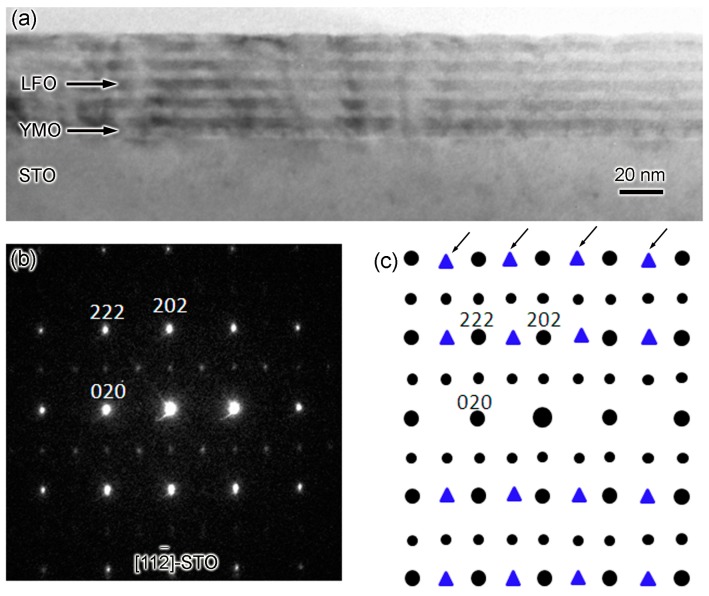
(**a**) The low-magnification TEM image of sample-III grown on (111)-STO substrate; (**b**) is the SAED pattern of sample-III with electron beam aligned along [11$\bar{2}$]-STO direction; (c) is the schematic of SAED pattern of Figure 6b. Black circles are the reflections observed in the experiment. Blue triangles (highlighted by black arrows) are the extra reflections if there are three types of domains.

Figure 6a shows the low-magnification TEM image of sample-III grown on (111)-STO substrate. Like samples I and II, the layer-by-layer morphology is observed. Here, the bright fringe is the YMO layer, and the black LFO layer. Figure 6b is SAED pattern taken with electron aligned along [1$1\bar{2}$]-direction of STO. The area used to take SAED pattern is highlighted by a white circle. By means of SAED pattern simulation [18], Figure 6c shows the schematic of SAED pattern of the LFO-YMO multilayers if there are three-type of domains. Large and small black circles are reflections observed in the experiment. Blue triangles are the extra reflections if there are three types of domain. Figure 6b,c suggest that there is only one type of domains in sample-III.

**Figure 7:**
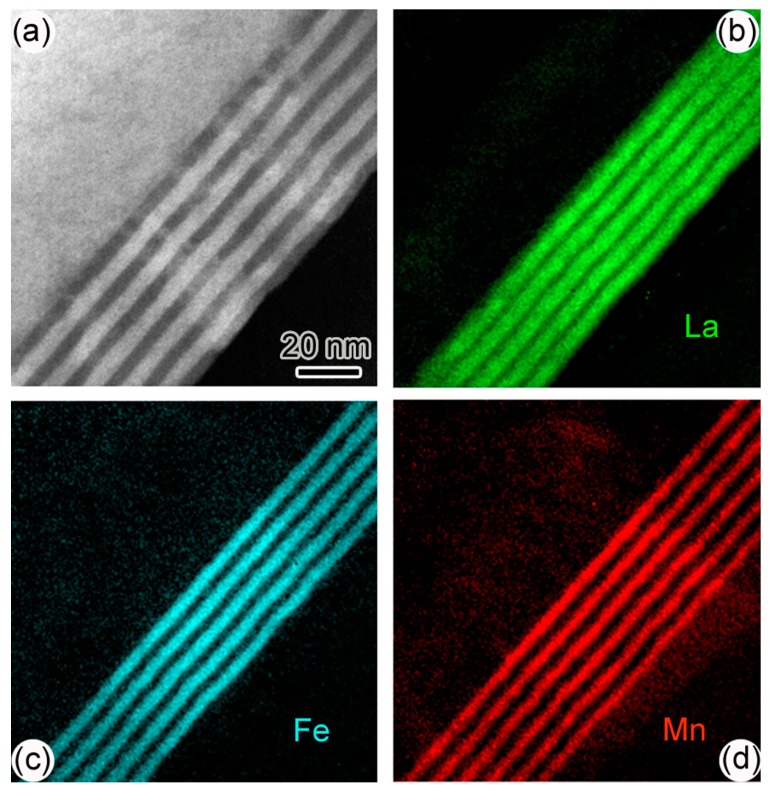
(**a**) Low-magnification HAADF image of sample-III; (**b**–**d**) are the element mapping images of La, Fe, and Mn in sample-III.

Figure 7a shows the low-magnification HAADF image of sample-III. The distinct layer structure is also observable. The bright layers are LFO and the dark ones YMO. The energy filtered TEM images of La, Fe, and Mn of sample-III are shown in Figure 7b–d, respectively. Obviously, LFO layers are separated from the YMO layers. Therefore, it can be inferred that the inter-diffusion between LFO and YMO layers is also not serious.

**Figure 8:**
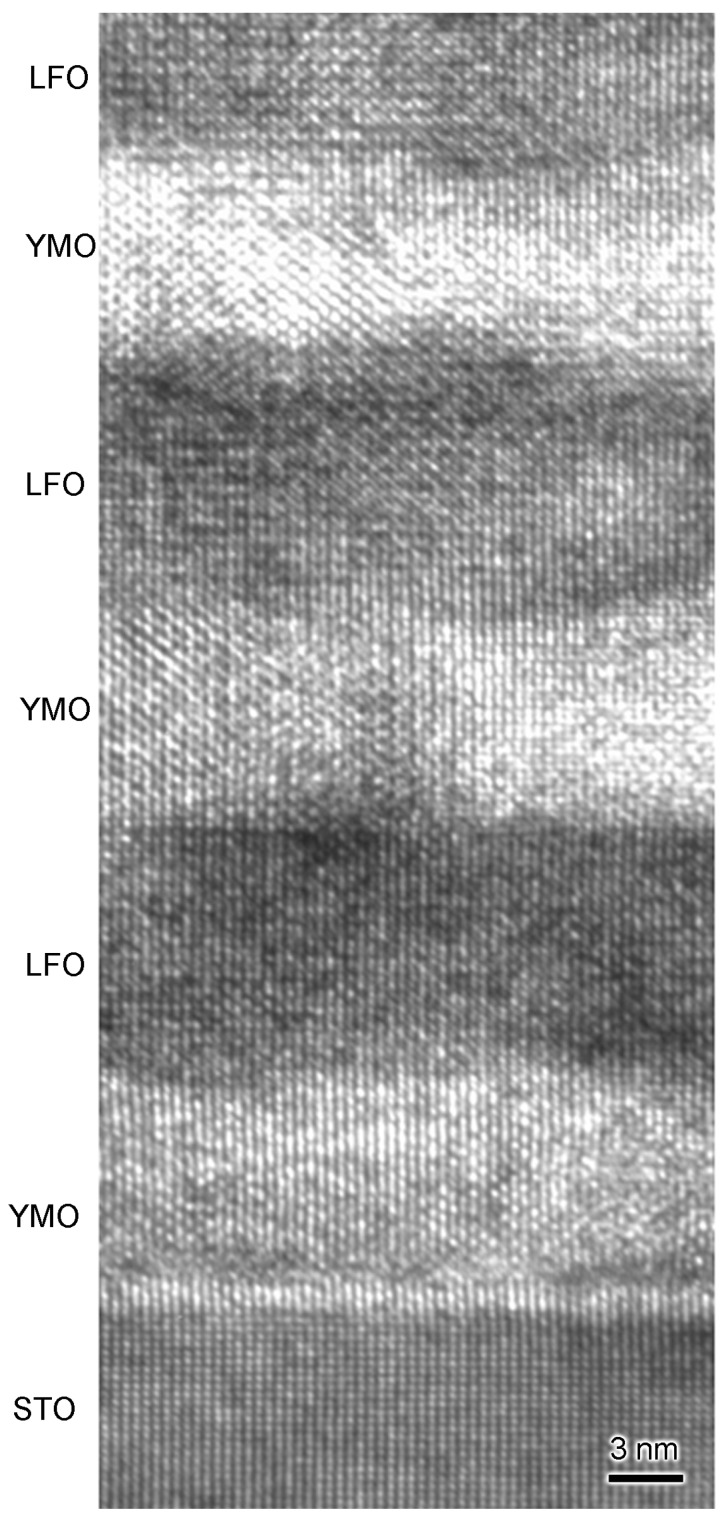
HRTEM image of sample-III. This image was taken with electron beam aligned along the [11-2]-STO zone axis.

A typical HRTEM image of sample-III, taken with electron-beam aligned along [11-2]-zone axis of STO, is shown in Figure 8. Apparently, LFO and YMO have quite good lattice fringes at the nanometer scale. The interface between LFO and YMO is also quite sharp. 

**Figure 9:**
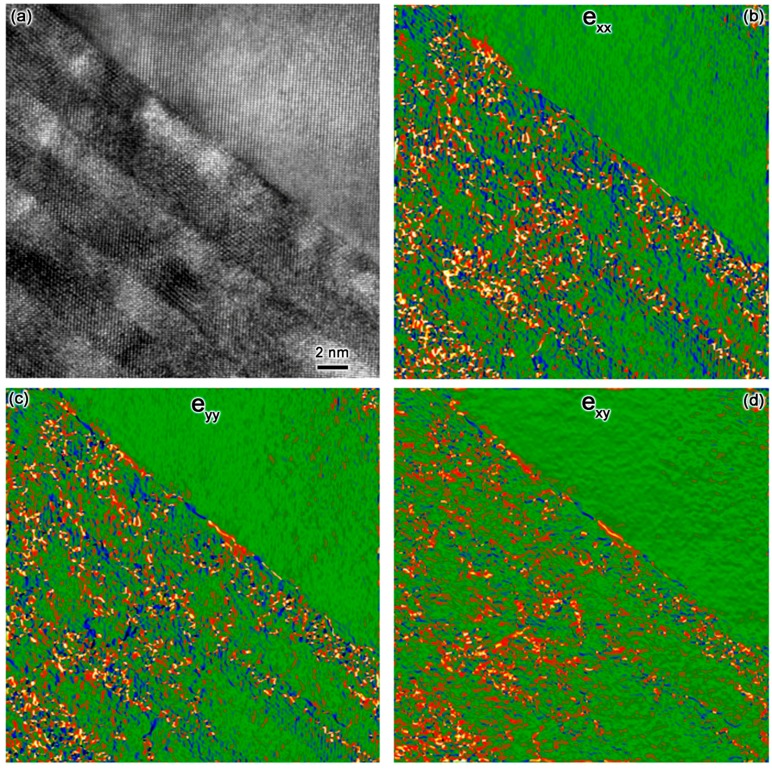
(**a**) A typical HRTEM image of sample-III; (**b**–**d**) are pure strain e_xx_ and e_yy_, as well as torsional strain e_xy_ (e_xx_ and e_yy_ are pure strain along the *x* and *y* axes, while e_xy_ is a torsional strain) that are extracted from geometric phase analysis, respectively.

The average strain state of LFO-YMO multilayers (sample-III) is studied by geometric-phase-analysis [19] and the results are depicted at Figure 9. Obviously, there are substantial strain e_xx_, e_yy_, and e_xy._ (e_xx_ and e_yy_ are pure strain along the *x* and *y* axes, while e_xy_ is a torsional strain [19]) in LFO-YMO multilayers. Accordingly, there is strong stress and resultant crystal defects in the LFO-YMO multilayers grown on (111)-STO substrate. This suggests that though there is only single-domain structure, strain relaxation and resultant crystal defects in sample III are universally existed. The reason for strain state is discussed below.

## 3. Discussion

**Figure 10:**
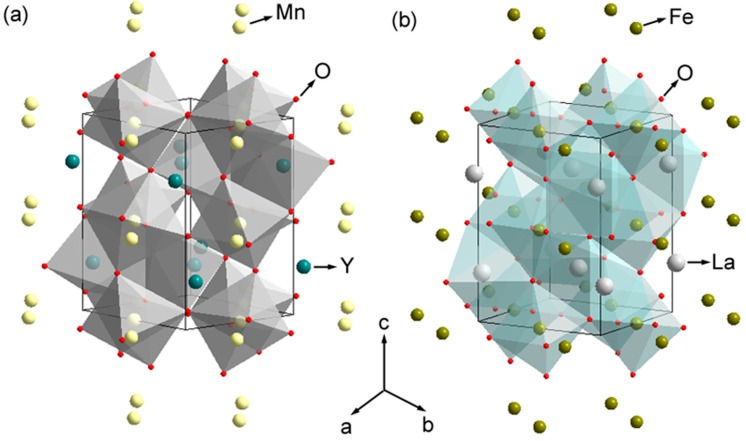
(**a**,**b**) are atomic structures of YMO and LFO, respectively. One can see the greater distortion of oxygen octahedra in YMO with respect to LFO.

We would like to qualitatively explain the formation of multi-domain structures in YMO-LFO multilayers grown on (001)-STO substrate from the viewpoint of mechanical strain. The lattice constants of orthorhombic LFO are: *a* = 5.556 Å, *b* = 5.565 Å, and *c* = 7.862 Å [20], and those of orthorhombic YMO are: *a* = 5.836 Å, *b* = 5.258 Å, and *c* = 7.357 Å [21]. STO has a simple cubic structure, *a* = 3.91 Å. It is noticed that *a* and *b* of LFO are quite close, but those of YMO are not; they are quite different. For the sake of simplicity, the orthorhombic perovskite can be viewed as pseudo-cubic structure. LFO can be treated as *a_P_* = 3.93 Å, while in YMO, *a_P_* = *b_P_* = 3.91 Å, and *c_P_* = 3.68 Å. In other words, LFO is closer to a cubic structure, but YMO is closer to a tetragonal one. Theoretically, the multi-domain structure is related to the relaxation of the mechanical strain among YMO, LFO, and STO [22,23]. SAED results (Figure 1b,c) prove that the in-plane orientation relationship between LFO and YMO are: [001]-[1-10]LFO // [110]-[1-10] YMO, [001]-[1-10]LFO // [110]-[001] YMO and [001]-[1-10]LFO // [001]-[1-10] YMOin the α-, β-, and γ-domain configurations, respectively. The in-plane lattice mismatch *ε* is defined as $\frac{\left(a1\;-\;a2\right)}{0.5\;\times \;\left(a1\;+\;a2\right)}$ [24] where *a*_1,2_ is the lattice constant of component layer 1 and 2 in the pseudo-cubic setting. Accordingly, the in-plane theoretical *ε* between STO and YMO (LFO), as well as YMO and LFO can be calculated and summarized in Table 2. Evidently, lattice mismatch *ε* between YMO and STO, LFO and YMO, can be as large as 6%. Theoretically, the large mismatch can be relaxed by the formation of crystal defects, such as domain structure, dislocations, and grain-boundaries. If we only consider the lattice mismatch between LFO and YMO, generation of α-domain should be dominated to minimize the strain energy of this system [22,23]. But it should be mentioned that in LFO-YMO multilayers, the YMO lattice has much more distorted oxygen octahedra than LFO (see Figure 10) [20,21]. In this case, *torsional torque* should be considered, too [22,23]. Thus, the multi-domain structure might be formed to relax the torsional strain besides shear strain [23], which has been discussed in the formation of multi-domain structures in ferroelectric thin films. This leads to the observation of the multi-domain structure in YMO-LFO multilayers grown on (001)-STO substrate. It should be mentioned that the existence of pure- and torsional-strain in YMO-LFO multilayers has been revealed by geometric-phase-analysis (see Figure 9).

When the substrate is changed to (111)-STO, the corresponding in-plane lattice mismatch *ε* in this system is outlined in Table 3. Apparently, the lattice mismatches between component layers are smaller than 3.5%, which is smaller than that in (001)-STO substrate. This may be the reason that there is only one type of domain when the substrate is (111)-STO.

Certainly, the above-discussion is qualitative. A quantitative method, like the first-principles calculation of formation energy in the different domain structures [25], should be applied to further explore the physical origin of the multi-domain formation.

Finally, it is valuable to mention that the above-mentioned domain structure characterization is very central to explaining the measured magnetic properties. Based on the above-mentioned microstructure analysis, the magnetic property of YMO-LFO multilayer grown on (111)-STO can be attributed to the ferrimagnetism generated at interface between YMO and LFO, since there is only a single domain. However, the ferromagnetic hysteresis loop measured in YMO-LFO multilayer grown on (001)-STO should be attributed to non-canceled multi-/twin-domain structure, rather than the designed interfaces. It has been reported that YMO films with multi-/twin-domain structures show the clear ferromagnetic hysteresis loop though YMO is an antiferromagnetic compound [26]. Considering the universal existence of multi-domain structure in perovskite thin films grown on (001)-substrates, the spin-glass behavior observed in the LaFeO_3_-LaMnO_3_ multilayer grown on (001)-STO substrate [10] may also come from the multi-domain structure.

## 4. Materials and Methods

Three LFO-YMO multilayers were grown on either (001)- or (111)-STO substrates (HeiFei Ke Jing Materials Technology Inc., Hefei, Anhui, China) by pulsed laser deposition (SKY Technology Development Inc., Shenyang, Liaoning, China). The details of growth process and corresponding parameters can be found in [9]. The TEM samples were prepared by mechanical grinding and ion-milling. The details of TEM sample preparation have also been reported previously [15]. The TEM characterization was conducted on a Tecnai F20 transmission electron microscope (FEI Inc., Hillsboro, OR, USA) equipped with a Gatan image filter system (Gatan Inc. Quantum 693 GIF system, Pleasanton, CA, USA) and a high-angle annual dark field (HAADF) detector (FEI Inc., Hillsboro, OR, USA). The energy filter TEM was conducted in TEM mode, and the characteristic EELS peaks for La, Fe, and Mn are 832, 710, 643 eV, respectively. The energy window with 20 eV is used to take energy filtered TEM. The energy dispersion in EELS experiment is 0.5 eV/channel. In the STEM experiment, the spot size of the electron probe is 2.0 Å and the collective angle of annual dark field ranges from 20 to 50 mrad. The convergence angle is set as 5 mrad. The TEM images were compiled by DigitalMicrograph (Gatan Inc., Pleasanton, CA, USA) and Photoshop.

## 5. Conclusions

The microstructure of LFO-YMO multilayers grown on (001)- and (111)-STO substrates is characterized by TEM. Detailed microstructure characterization manifests that there is mainly a multi-/twin-domain structure in LFO-YMO multilayers grown on (001)-STO, and only a single-domain structure on (111)-STO substrate. The formation of multi-/twin- and single-domain structures in YMO-LFO multilayers can be qualitatively explained by the lattice mismatch in this system. The characterization of domain structure in multilayers is of great importance to understanding ferrimagnetism in YMO-LFO multilayers. 

## Figures and Tables

**Table 1:** The sample-parameters and synthesis conditions of three LaFeO_3_-YMnO_3_ (LFO-YMO) multilayers (samples I–III) characterized by transmission electron microscopy in this work. The growth time column shows the deposition time of LFO and YMO layers. It should be mentioned that we used the growth time to control the thicknesses of YMO and LFO in multilayers. STO represents SrTiO_3_.

Sample ID	Periodicity Thickness (nm)	Growth Time (Second)	Periodicity Number	Grown Substrate	Growth Temperature (°C)	Background Pressure (Pa)	Growth Oxygen Pressure (Pa)
I	22.0	300/300	10	(001)-STO	780	3 × 10^−^^5^	30
II	4.0	30/30	20	(001)-STO	780	3 × 10^−^^5^	30
III	8.0	90/90	5	(111)-STO	780	3 × 10^−^^5^	30

**Table 2:** Theoretical lattice mismatch ε, between component compounds, along two orthogonal in-plane directions when the substrate is (001)-STO. N. A represents not applied.

Configuration	YMO (002)	YMO (110)	LFO (001)
STO (001)	0.13%; 0.13 %	0.13%; −6.0%	0.66%; 0.66%
LFO (001)	−0.56%; −0.56%	−0.56%; −6.6%	N. A

**Table 3:** Theoretical lattice mismatch ε, between component compounds, along two orthogonal in-plane directions when the substrate is (111)-STO. Two orthogonal in-plane directions are [1-10] and [11-2]. In this table, lattice mismatch is calculated by assuming lattice constants of YMO and LFO in pseudo-cubic setting. The lattice constants of LFO and YMO in pseudo-cubic setting are 3.93 Å and 3.83 Å, respectively. N. A represents not applied.

Configuration	YMO (111)	LFO (111)
STO (111)	−2.55%; −2.79%	−0.63%; 0.69%
LFO (111)	−3.17%; −3.48%	N. A
